# The Pathological Relations of the Gastric and Intestinal Tubules

**Published:** 1871-05

**Authors:** Austin Flint

**Affiliations:** Professor of Practice of Medicine in Bellevue Hospital Medical College


### THE PATHOLOGICAL RELATIONS OF THE GASTRIC AND INTESTINAL TUBULES.

A Lecture by AUSTIN FLINT, M.D., Professor of Practice of Medicine in Bellevue Hospital Medical College.

Mr. President:—The paper which I am about to read will not contain any original observations, experience in practical microscopy being necessary, and I have not the qualifications or the leisure to engage in it. As regards the histological aspect of my subject, the facts to which I shall refer have been contributed by the English observers, Handfield Jones, Wilson Fox, and Samuel Fenwick.

I have for years been convinced that the secretory glands of the alimentary canal form a domain in pathology unexplored, but from which important accessions are to come, and I have predicted that the tubules there found will present degenerative changes, which will satisfactorily account for that class of cases characterized by progressive and fatal inanition. I shall first give a history of some cases of this class which have fallen under my own observation.

Case I.—Gentleman, æt. 60. Health good up to the time anorexia began. No disease could be detected in any of the organs after a careful investigation, but for all, his appetite continued to fail till he could scarcely be prevailed on to take food in any form. His weight failed without notable emaciation, and by degrees his strength so far yielded that he was obliged to keep to his bed. At the time I saw him the pulse and skin did not denote fever. Various tonics and stimulants were given, but with no result.

Inanition continued and patient died. There was no autopsy.

I studied the case carefully, and was unable to account for the nature and seat of the disease.

Case II.—In March, 1866, saw in consultation a gentleman æt. 60, who had been ailing some months before I saw him. Without any appreciable cause his strength began to fail as anorexia progressed.

Shortly after I saw him he died. As in the former case, no cause could be discovered. An autopsy was refused by the friends.

Case III.—In the summer of 1868, I saw often with my lamented colleague, Dr. Geo. Elliott, a gentleman æt. about 55 years. The history of this case only differed from the others in the fact that he had occasional vomiting. Toward the close he passed under the hands of an homœopath; but I have learned that he died by exhaustion.

CASE IV.—In May, 1869, an analagous case came under my observation in the practice of Dr. Jno. P. Hutchinson, of Brooklyn. Dr. Willard Parker was associated in consultation. The age of the patient was 60. He had been ailing for several months. The only difference in this case from the others was that he had occasional diarrhœa, but the history and symptoms denoted intestinal indigestion, and not ulceration or inflammation. Emaciation was more marked in this than in the previous cases.

The cases here recorded are males, but I visited, with my colleague, Dr. Fordyce Barker, a female patient in October, 1868, and another in January, 1869, the facts in both being similar to those I have given a summary of. The ages of each of Dr. B.'s cases were in the neighborhood of 60. It may have occurred to some present that there is a relation between the foregoing class of cases and the "*idiopathic anœmia*" of Addison.

Dr. Addison says: "For a long period I have from time to time met with a very remarkable form of general anæmia without any discoverable cause—cases in which there had been no previous loss of blood; no exhausting diarrhœa; no chlorosis; no purpura; no renal affection, splenic miasmata, glandular, strumous or malignant disease. * * * * It was while seeking in vain to throw some additional light on this form of anæmia that I stumbled on the curious facts that it is my more immediate object now to make known to the profession."

The facts referred to at the close of the quotation are the existence in these cases of infra-renal disease, and the absence of lesion elsewhere, to account for the anæmia and melasma. It is a reasonable supposition that Addison's cases, with and without the melasma, are to be classed with those which I have recited. Addison did not seek for the disease in the gastro-intestinal tubules. It is not a recent conjecture of mine that, had his microscopical researches taken this direction, he would have found a more satisfactory explanation of the infra-renal capsules on which, as he says, he stumbled.

In a published clinical lecture, in 1860, I said: "I have not the presumption to explain these cases, but I suspect there exists degenerative disease of the glandular tubuli of the stomach," and, I added, "I shall be ready to claim the merit of the idea if the laborious researches of some one shows it to be correct."

Assuming that the cases of Addison's "Idiopathic Anæmia"

exemplify the pathological relations of the gastro-intestinal tubules, an important fact is that the anæmia is sometimes a remarkably prominent feature. In 1859, I saw a case at the New Orleans Charity Hospital, but as I was not aware at that time that Addison attributed the anæmia, when not accompanied by melasma, to supra-renal disease, the kidneys were not examined. The patient was a Spaniard, æt. 30; emaciated and anæmic, with no definite ailments except loss of appetite, occasional diarrhœa, and hemorrhoids. The hemorrhoids were removed, but no improvement in the general symptoms took place; a thorough examination of the different organs showed negative results. By degrees patient grew weaker and weaker, and died.

*Post mortem* examination gave no disease of lungs, heart, stomach, or intestines. The spleen was somewhat enlarged. Other organs not examined. I added a postscript, stating that the blood was not examined for leucocythæmia.

In 1860, I saw another case very similar, but no autopsy was obtained.

Greenhow has tabulated 196 cases of disease of supra-renal capsules of whatever kind, and of bronzed skin, with or without supra-renal disease.

Without entering into a discussion on the topic, I will simply remark that to prove the dependence of the symptoms on lesions of the gastric intestinal tubules, would not disprove a pathological relation of some kind in certain cases, between these lesions or their immediate effects and disease of the supra-renal capsules. The testimony of pathological anatomy yet remains to prove the views advanced, and an important contribution has been made in this quarter by Dr. Samuel Fenwick, of London, who has demonstrated extensive and destructive lesions of the gastric tubules, in a well-marked case belonging to the class which I have considered. In the *London Lancet*, of July 16th, 1870, he gives the case in full. At the *post mortem*, the stomach was found empty, excepting a small quantity of gas; here were no signs of *post mortem* digestion. When placed beneath the microscope the pits on the surface of the membrane were rather larger than usual, and well defined. The whole of the glandular structure of the organ was in a state of atrophy, and in no part could a section of normal tissue be found. In the pyloric and middle regions the secreting tubes seemed to be converted into a mass of connective tissue, but toward the cardiac region there is a trace of glandular structure. Here the tubes were scattered, flask like, and filled with granular matter and fatty epithelial cells. In other places the ends of the tubes were expanded. Brunner's glands were unusually large. The villi of the upper part of the intestine were large, prominent, and contained fat, not as an emulsion, but as large drops in the interior of the villi.

In 1854, Handfield Jones wrote a paper on morbid changes in the mucous membrane of the stomach, and reported 100 cases where it had been examined with different diseases of the system. Of these, 28 were normal or nearly so, 72 diseased—and of these, 25 presented different conditions of diseased tubules.

Dr. Wilson Fox, in 1858, published the accounts of 100 cases taken indiscriminately, and finds different conditions of disease, but does not give the proportions nor particulars.

Dr. Fenwick says, in his researches in cancer, that out of 100 cases, exclusive of cancer, he found 17 in which there were extensive tubular changes in the splenic and middle region of the stomach, and in 75 of cancer of the breast, the same regions were extensively diseased. In twelve per cent. of cancer of uterus there were found morbid changes, and in ten of eighteen cases of cancer the tubules were more or less diseased. In conclusion, Dr. F. says, that it may readily be seen that in these changes there is an explanation of the anæmia which so often accompanies malignant disease.

Dr. Fenwick, in 1864, examined into the state of the tubules of the stomach in scarlatina. Of ten cases in which death took place during the first week, the tubules were greatly distended by granular and fatty matter, or by small cells intermixed with granules. Of six cases where death took place from the second to the third week the tubes were less distended, their closed ends being still loaded with granular matter. Dr. Murchison has examined the stomach of twenty cases in this disease, and noted similar results, but they did not occur in every case, and he has found them also in other diseases.

Dr. F. also examined microscopically the vomited matters in the third week of scarlatina, and found them to contain fibrinous casts of the stomach tubes.

In accordance with the propositions at the outset, I shall consider a few points in the pathological relations of the gastric and intestinal tubules in different diseases. It is frequently difficult to account for death in different forms of disease otherwise than to suppose that these glands were diseased. In phthisis how common it is to see those whose lungs are extensively destroyed live for years, whilst on the other hand the reverse takes place. We account for the fact by saying there is or is not a tolerance of the disease, but we do not explain. But clinical experience shows, other things being equal, that this rests on the ability to injest or digest food, and as has been shown by observations of Fox, Jones, and Fenwick, that the tubules in chronic diseases are more or less diseased, it is not unfair to assume that in one class of cases extensive change has taken place, and the other not so much, if any.

Reasoning from a physiological, clinical, and pathological standpoint, we are warranted in the view that impairment of the digestive organs, together with anorexia, is symptomatic of morbid change in the gastric and intestinal tubules, but it remains to be determined whether the mobility to take certain kinds of food may not have a relation to the destruction of the gastric and intestinal tubules separately.

In writing this paper, my object has been to make further investigation on the subject. It forms a territory in pathology but slightly explored. I shall be glad if my remarks induce any to prosecute studies in this direction.—*Medical and Surgical Reporter.*

